# Developing “Adulting for Health”: Investigating the Health Needs of Neurodivergent Emerging Adults

**DOI:** 10.7759/cureus.41102

**Published:** 2023-06-28

**Authors:** Emily Hotez, Julianna A Rava, Lindsay Shea, Alice Kuo

**Affiliations:** 1 General Internal Medicine, University of California Los Angeles David Geffen School of Medicine, Los Angeles, USA; 2 Public Health, University of California Los Angeles Fielding School of Public Health, Los Angeles, USA; 3 Health Policy, Drexel University AJ Drexel Autism Institute, Philadelphia, USA; 4 Division of Medicine-Pediatrics, University of California Los Angeles David Geffen School of Medicine, Los Angeles, USA

**Keywords:** preliminary, pilot project, child and youth mental health, emerging adulthood, neurodiversity

## Abstract

Introduction: Neurodivergent emerging adults - defined as individuals between the ages of 18 and 30 with intellectual and/or developmental disabilities (e.g., attention-deficit hyperactivity disorder (ADHD), autism, cerebral palsy, learning disabilities, seizures, developmental delays, with or without intellectual impairment) and physical and/or sensory disabilities (e.g., blindness or hearing impairment) - experience poor mental and physical health outcomes. Existing interventions are insufficient because they are not based on the self-reported and developmental needs of this population.

Methods: The current study is an exploratory pilot study that features a multidimensional health-based needs assessment of self-identified neurodivergent emerging adults with ADHD, learning disabilities, autism, and other conditions, mean (M) age = 22.8; standard deviation (SD) = 3.4; n = 26). This research used validated measures. The assessment - administered via Qualtrics to the participants in two sites - included the Mental Health Continuum-Short Form, Kessler-6 Psychological Distress Scale, Project EAT (Eating and Activity over Time)-IV (with the intuitive eating, weight-related control, emotional eating, and physical activity subscales), and an original health-focused needs assessment developed by interdisciplinary healthcare professionals and neurodivergent individuals.

Results: The sample reported low positive mental health, with only 3% reportedly "flourishing." The sample also reported high psychological distress according to clinical and psychometric cut-off scores; varied intuitive eating and weight-control behaviors and attitudes; and distinct needs related to integrating the principles of health promotion into daily life, navigating the healthcare system, and learning from healthcare professionals. Based on these findings, we present an initial conceptualization of “Adulting for Health,” a potential virtual education program to promote health-related knowledge and capacities for this population.

Conclusions: The results from this exploratory pilot study can be incorporated into existing programs and spur efforts to develop and test new interventions that can ameliorate health disparities for neurodivergent emerging adults.

## Introduction

Emerging adulthood (ages 18-30) is a developmental period characterized by transitions and instability in healthcare, education, employment, and interpersonal domains [1,2]. Emerging adults experience distinct health challenges relative to adolescent and older adult populations, including increased rates of depression, anxiety, eating-related disorders, substance use, non-suicidal self-injury, suicidal ideation, and suicide attempts.They also experience fluctuations in their positive mental health, defined by a sense of meaning, purpose, and connection [2]. Several physical health issues are particularly emphasized in the literature, such as obesity, hypertension, and pre-diabetes, which compound to create additional mental health challenges [3]. These health issues often permeate into later adulthood [4].

The aforementioned health challenges are due to a range of factors, including gaps in the healthcare transition processes required for moving from a child to an adult model of healthcare with or without a transfer to a new clinician [5]. Indeed, US emerging adults and their caregivers are not receiving adequate transition preparation [5,6]. There are multiple cascading effects of this shortcoming, including unaddressed health issues [5]. In addition to healthcare service fragmentation, poor health outcomes are exacerbated by emerging adults’ challenges implementing health-promoting lifestyle behaviors. Indeed, they often have poor diet [7], engage in insufficient physical activity [8], and demonstrate low treatment adherence and access [1,7,9] due to both individual and systemic factors.

The challenges faced by neurodivergent emerging adults

Health disparities are particularly pronounced for neurodivergent emerging adults, including those with intellectual and/or developmental disabilities (e.g., attention-deficit hyperactivity disorder (ADHD), autism spectrum disorder, cerebral palsy, learning disabilities, seizures, developmental delays, with or without intellectual impairment), physical disabilities, and chronic conditions (e.g., blindness or hearing impairment). This population experiences lifelong issues related to cardiovascular, gastrointestinal, metabolic, and mental health, as well as lower overall health and life expectancy [10-13]. Although there is limited research on neurodivergent emerging adults’ positive or “flourishing” mental health, their challenges are well-documented [14]. Health challenges are due to genetic susceptibility, challenges with implementing health-promoting lifestyle behaviors, psychotropic medication use, and lack of physician training and education focused on this population [15-18].

Opportunities to promote the health of neurodivergent emerging adults

Although emerging adulthood can represent a developmental period of risk, it also represents a key opportunity for health-promoting interventions. Sensitive periods of development have traditionally been conceptualized as occurring in early childhood, yet contemporary developmental science suggests that periods of transitions and instability can represent pivotal opportunities for intervention [19,20]. Emerging adults’ burgeoning independence and multiple life transitions make this period a particularly optimal time for multiple points of intervention.

Increasing recognition of this opportunity has spurred the development of “adulting” interventions: interventions that seek to cultivate skills and capacities necessary for “adulting.” “Adulting" is a colloquialism often used by emerging adults in popular culture and is defined as engaging in tasks and activities that conventionally accompany the increased responsibilities of adulthood. To be sure, developing programming aligned with cultural language and values of the target population may help ensure that the intervention is tailored toward the needs of potential participants, yet the vast majority of “adulting” programs do not specifically target health-related skills and capacities that are important in adulthood [21]. Rather, they adapt a more generalized approach focused on a range of topics (e.g., finances, vocation, and daily living) with less emphasis on health [21]. When they do focus on health, they primarily focus on promoting general education surrounding nutrition or mental health or are geared toward the general college student population, rather than focus on the health of neurodivergent populations [22].

There is, in effect, a paucity of neurodiversity-oriented health-based interventions for emerging adults. Thus, existing approaches are not necessarily attuned to the unique experiences, priorities, and preferences of this population [23-25]. Pioneering approaches in this area include the Academic Autistic Spectrum Partnership in Research and Education (AASPIRE) toolkit: a widely used healthcare resource to support autistic individuals as they transition to adult care, developed from interviews with autistic individuals [26]. The AASPIRE work highlights the utility of basing health-focused interventions for this population on the input from neurodivergent emerging adults themselves. Taken together with research that emphasizes the utility and acceptability of virtual approaches led by interdisciplinary health teams [27], there is a necessity for needs assessment research with neurodivergent emerging adults to inform virtual health-based programming led by diverse healthcare professionals.

Based on the gaps in the field and the opportunities inherent in creating virtual interventions for neurodivergent emerging adults, there is a need for research that directly translates into the development of scalable practices and approaches to promote health for this population. The current study addresses these gaps.

The current study

We conducted an exploratory pilot study that aimed to 1) assess the mental and physical health challenges and experiences of neurodivergent emerging adults; 2) determine their self-reported a) health and healthcare needs and b) learning preferences; and 3) conceptualize a preliminary “Adulting for Health” curriculum, to be pilot-tested in subsequent studies based on the findings of the present study.

## Materials and methods

Sample

The sample was recruited through two sites. Site 1 was a continuing adult education office serving neurodivergent individuals transitioning out of high school into work or school, as well as those currently in college or employed part or full time. Site 2 was a four-year public university with a professional development program serving neurodivergent individuals auditing a class on emerging adulthood. Recruitment efforts were conducted via e-mail.

The sample characteristics are reported in Table 1. The sample included 26 neurodivergent emerging adults (mean (*M*) age = 22.8, SD = 3.4) with a range of self-reported neurodivergent conditions (ADHD: *n* = 12, 46%; other conditions: *n* = 6, 23%; chronic illness: *n* = 4, 15%; learning disability: *n* = 4, 15%; autism: *n* = 3, 12%; and physical disability: *n* = 2, 8%). Almost one-fifth (*n* = 5, 19%) reported more than one disability. The sample was diverse with respect to race (White: *n* = 13, 50%; Black/African American: *n* = 4, 15%; Asian: *n* = 2, 8%; others: *n* = 2, 8%; and Native American/Alaska Native: *n* = 1, 4%) and ethnicity (Hispanic: *n* = 7, 27%). Approximately half identified as female (*n* = 13, 50%), with the remainder of the participants identifying as male (*n* = 8, 31%) or non-binary (*n* = 3, 12%). Approximately half reported the sexual identity of straight (*n* = 13, 50%), with the remainder of the participants identifying as bisexual/pansexual (*n *= 6, 23%), gay/lesbian (*n* = 1, 4%), and other sexual identities (*n* = 4, 15%).

**Table 1:** Demographics of the participants Three Site 1 participants were employed part or full time, and one was enrolled in college part or full time. All Site 2 participants were enrolled in college part or full time. SD: standard deviation; ADHD: attention deficit hyperactivity disorder

	Site 1		Site 2		Total	
	(*n* = 7)		(*n* = 19)		(*n* = 26)	
Characteristic	n	%	n	%	n	%
Age						
18-20	0	0%	7	37%	7	27%
21-23	1	14%	9	47%	10	39%
24-26	0	0%	2	11%	2	8%
27-29	1	14%	0	0%	1	4%
30+	1	14%	0	0%	1	4%
Missing	4	57%	1	5%	5	19%
Mean (SD)	22.8 (3.4)					
Race						
White	3	43%	10	53%	13	50%
Black or African American	2	29%	2	11%	4	15%
Native American or Alaska Native	0	0%	1	5%	1	4%
Asian	0	0%	2	11%	2	8%
Different race	0	0%	2	11%	2	8%
Prefer not to say	0	0%	1	5%	1	4%
Missing	2	29%	1	5%	3	12%
Hispanic						
Yes	0	0%	7	37%	7	27%
No	5	71%	12	63%	17	65%
Missing	2	29%	0	0%	2	8%
Gender						
Male	1	14%	7	37%	8	31%
Female	3	43%	10	53%	13	50%
Not listed / others / prefer not to say	1	14%	2	11%	3	12%
Missing	2	29%	0	0%	2	8%
Sexual identity						
Bisexual or pansexual	1	14%	5	26%	6	23%
Gay or lesbian	1	14%	0	0%	1	4%
Straight	2	29%	11	58%	13	50%
Prefer not to say / others	1	14%	3	16%	4	15%
Missing	2	29%	0	0%	2	8%
Disabilities (select all that apply)						
Learning disability	3	43%	1	5%	4	15%
ADHD	2	29%	10	53%	12	46%
Autism	1	14%	2	11%	3	12%
Physical disability	0	0%	2	11%	2	8%
Chronic illness	1	14%	3	16%	4	15%
Others	0	0%	6	32%	6	23%
Multiple	2	8%	3	12%	5	19%
Missing	2	29%	0	0%	2	8%

Procedure

All data collection efforts were conducted via Qualtrics and occurred between January and April 2022. Data collection featured a mental health assessment on positive mental health and psychological distress, a physical health assessment on eating-related attitudes and behaviors and physical activity, and a survey on self-reported health needs and learning preferences. Survey development included consultations with healthcare professionals affiliated with Site 1 and 2 and neurodivergent researchers. The authors gleaned informal feedback and suggestions from neurodivergent and neurotypical emerging adults enrolled in a four-year public university to ensure item clarity and relevance. All data collection efforts prioritized best practices of accessibility (e.g., Likert scales were conveyed utilizing both written and graphical cues) [28]. 

This research was approved by the University of California, Los Angeles (UCLA) Institutional Review Board (IRB), with IRB approval number 20-000320.

Measures

Mental Health

Assessments included the Kessler-6 (K6) Psychological Distress Scale [29] and the Mental Health Continuum-Short Form (MHC-SF) [30]. The K6 is a widely used and well-validated scale examining self-reported emotional distress in the past 30 days. It is extensively used to identify individuals at high risks for severe mental illness without a clinical diagnosis [29]. The responses to the six K6 assessment items were on a five-point scale; scores were then summed (range of 0-24). A score of 13 or greater has been found to indicate a clinically significant degree of emotional distress and is an established cut-point for the K6 [29]. In the current study, the K6 demonstrated strong internal consistency (Cronbach’s alpha = 0.84).

The MHC-SF measures positive mental health or “flourishing” and is comprised of 14 items, representing various feelings of positive mental health. The respondents rate the frequency of each feeling in the past month on a six-point Likert scale. The MHC-SF contains three subscales, each of which demonstrated strong internal consistency in the current study: 1) emotional well-being (Cronbach’s alpha = 0.81), 2) psychological well-being (Cronbach’s alpha = 0.84), and 3) social well-being (Cronbach’s alpha = 0.85). The respondents were classified as “flourishing” if they reported that they experience “every day” or “almost every day” at least seven of the feelings, with at least one from the emotional well-being subscale [30].

Physical Health

Physical health domains were non-exhaustive but selected based on physical health challenges experienced by the general emerging adult population that are emphasized in the literature. Physical health was assessed by utilizing three scales created for the fourth wave of Project Eating and Activity over Time (EAT) [31]. Scales included intuitive eating (Cronbach’s alpha = 0.88), weight-related concern (Cronbach’s alpha = 0.76), and emotional eating (Cronbach’s alpha = 0.95). Project EAT-IV physical activity questions were also included; the participants were asked how frequently they engage in strenuous, moderate, and mild exercise, as well as their reasons for engaging in physical activities.

Self-Reported Health Needs

Self-reported health needs were determined via original questions on 1) the confidence in health-related knowledge abilities and 2) desire to learn more about each of these topics. Specific topics were based on a consensus among interdisciplinary healthcare professionals, neurodivergent researchers, and previous research. Areas of high need were identified based on whether 50% or more reported confidence in one of the lower three categories (not at all, a little, or somewhat) and 50% or more reported that they agreed or strongly agreed that they wanted to learn more about a topic. The participants were also asked about knowledge sources and learning preferences.

## Results

Mental and physical health

Table 2 reports the descriptive statistics on mental health indicators. With respect to positive mental health, the emotional well-being mean was 10.8 (SD = 2.4), the social well-being mean was 12.2 (SD = 5.7), and the psychological well-being mean was 20.9 (SD = 5.6). The total mean score was 43.9 (SD = 11.3), and the total number of respondents who were categorized as “flourishing” was three (12%). With respect to the K6, the mean score was 14.2 (SD = 5.3). Applying K6 cut-offs determined through previous research [29], the mean K6 (greater than or equal to 13) demonstrates that most participants reported experiencing high distress over the past 30 days.

**Table 2:** Mental and physical health assessments ^1^Total “flourishing” count: *n* = 3 (12%). ^2^Intuitive eating quartiles: 1 (19%); 2 (27%); 3 (15%); 4 (15%). ^3^Weight-related concern quartiles: 1 (19%); 2 (19%); 3 (19%); 4 (15%). ^4^Emotional eating quartiles: 1 (19%); 2 (23%); 3 (19%); 4 (12%) MHC-SF: Mental Health Continuum-Short Form; K6: Kessler-6 Psychological Distress Scale; EAT-IV: Eating and Activity over Time-IV; SD: standard deviation

Health indicator (assessment)	Mean	SD
Positive mental health (MHC-SF)^1^	43.9	11.3
Emotional well-being	10.8	2.4
Social well-being	12.2	5.7
Psychological well-being	20.9	5.6
Psychological distress (K6)	14.2	5.3
Eating-related behaviors and attitudes (EAT-IV)		
Intuitive eating^2^	20.9	4.3
Weight-related concerns^3^	10.9	3.9
Emotional eating^4^	11.6	5.5

Table 2 reports descriptive statistics on eating-related behaviors and attitudes. With respect to intuitive eating, the mean score was 20.9 (SD = 4.3). The respondents were distributed among the four quartiles (Q1: 19%; Q2: 27%; Q3: 15%; Q4: 15%), with a greater proportion below the median (46%). With respect to weight-related concerns, the mean score was 10.9 (SD = 3.9). The respondents were distributed approximately equally among the four quartiles (Q1: 19%; Q2: 19%; Q3: 19%: Q4: 15%). With respect to emotional eating, the mean score was 11.6 (SD = 5.5). The respondents were distributed among the four quartiles (Q1: 19%; Q2: 23%; Q3: 19%; Q4: 12%), with a greater proportion below the median (42%).

Table 3 reports descriptive statistics on physical activities. Almost one-third reported engaging in strenuous physical activities for at least 30 minutes to two hours per week (in alignment with federal guidelines that suggest 75 minutes per week of exercise [32] (*n* = 8, 31%). Moreover, more than half reported this frequency for moderate exercise (*n* = 15, 58 %) and mild exercise (*n* = 15, 58%). The most common exercise reason was “to be healthy” (*n* = 14, 54%), followed by “because it makes me feel good” (*n* = 12, 46%), “to build up my strength” (*n* = 10, 38%), “to spend time doing an activity I enjoy” (*n* = 9, 35%), “to avoid health problems" (*n* = 8, 31%), and “to lose weight or look a certain way” (*n* = 8, 31%).

**Table 3:** Physical activity assessments ^1^Missing *n* = 7-8 EAT-IV: Eating and Activity over Time-IV

Physical activity in the past month (EAT-IV):^1^	None-<30 min/week n (%)	30 min-2 hours/week n (%)	2.5-4 hours/week n (%)	4.5+ hours/week n (%)
Strenuous exercise (e.g., biking fast, aerobics, jogging)	11 (42%)	3 (12%)	1 (4%)	4 (15%)
Moderate exercise (e.g., walking quickly, easy bicycling)	4 (15%)	7 (27%)	3 (12%)	5 (9%)
Mild exercise (e.g., walking slowly, bowling)	4 (15%)	6 (23%)	5 (19%)	4 (15%)

Self-reported health needs

Table 4 reports confidence in and desire to learn more about specific health-related knowledge and abilities. Areas of high need included capacities related to being able to 1) discuss different health insurance coverage options, 2) find out details about their health insurance and ask questions, 3) find a doctor, 4) buy and cook healthy food on a budget, 5) practice healthy nutrition, and 6) get a healthy amount of sleep.

**Table 4:** Needs assessment ^1^Missing: *n* = 2 Bold texts are areas of high need (50% or more = reported not at all, a little, or somewhat confident; 50% or more = agreed or strongly agreed they wanted to learn about it).

	I feel confident in my ability to^1^											
	Not at all		A little		Somewhat		Very		Extremely		N/A	
Topics	n	%	n	%	n	%	n	%	n	%	n	%
Find a mental health provider	4	15%	1	4%	6	23%	6	23%	5	19%	2	8%
Discuss substance use with my medical provider	1	4%	2	8%	4	15%	10	39%	3	12%	3	12%
Manage stressful emotions	1	4%	2	8%	9	35%	11	42%	1	4%	0	0%
Navigate the healthcare services that I need	3	12%	3	12%	6	23%	8	31%	3	12%	1	4%
Discuss the different health insurance coverage options	6	23%	6	23%	6	23%	3	12%	2	8%	1	4%
Find out details about my health insurance and ask questions	4	15%	11	42%	4	15%	3	12%	2	8%	0	0%
Find a doctor	4	15%	2	8%	9	35%	5	19%	4	15%	0	0%
Make a doctor’s appointment	3	12%	2	8%	3	12%	6	23%	8	31%	1	4%
Prepare for my doctor’s visits	2	8%	2	8%	3	12%	9	35%	6	23%	2	8%
Manage my healthcare	1	4%	5	19%	5	19%	8	31%	3	12%	1	4%
Manage my medication	2	8%	2	8%	8	31%	8	31%	3	12%	4	15%
Advocate for myself as a patient	2	8%	3	12%	3	12%	6	23%	9	35%	1	4%
Know which foods are nutritious / healthy	0	0%	2	8%	7	27%	9	35%	6	23%	0	0%
Buy and cook healthy food on a budget	1	4%	4	15%	8	31%	7	27%	4	15%	0	0%
Practice healthy nutrition	0	0%	3	12%	10	39%	7	27%	4	15%	0	0%
Engage in physical activities	0	0%	2	8%	6	23%	11	42%	5	19%	0	0%
Get a healthy amount of sleep	4	15%	2	8%	7	27%	8	31%	3	12%	0	0%

The participants’ existing healthcare knowledge and skills were obtained from a range of sources, including parents (*n* = 21, 81%), doctors and healthcare professions (*n* = 10, 38%), friends and peers (*n* = 6, 23%), schools and teachers (*n* = 5, 19%), social media and news (*n* = 4, 15%), other family members (*n* = 3, 12%), and on their own (*n* = 3, 12%). The participants’ existing health knowledge and skills were obtained from parents (*n* = 18, 69%), social media and news (*n* = 13, 50%), doctors and healthcare professionals (*n* = 11, 42%), schools and teachers (*n* = 10, 38%), friends and peers (*n* = 9, 35%), other family members (*n* = 5, 19%), and on their own (*n* = 4, 15%).

The participants preferred a range of virtual accommodations and features, including having a chat option (*n* = 13, 50%), having an agenda in advance (*n* = 9, 35%), having a question and answer (Q&A) session at the end of the class (*n* = 7, 27%), having the presenter’s slide deck (*n *= 7, 27%), and others, including discussion-based activities and opportunities for note-taking (*n* = 5, 19%).

These findings - in combination with previous research results - coalesced in a draft curriculum for “Adulting for Health” (see Appendix).

## Discussion

The current study aimed to 1) assess the mental and physical health challenges and experiences of neurodivergent emerging adults; 2) determine their self-reported a) health and healthcare needs and b) learning preferences; and 3) conceptualize a preliminary “Adulting for Health” curriculum to be pilot-tested in subsequent studies. This study addresses the need to support the health and well-being of neurodivergent emerging adults and fills critical gaps surrounding a lack of interventions based on the self-identified needs of this population. This research has several key findings that coalesced into several recommendations for “Adulting for Health.”

Mental and physical health

Few neurodivergent emerging adults in our sample were flourishing (3%). To date, there is limited research on neurodivergent emerging adults’ flourishing, with greater attention paid to underscoring their challenges [14]. Additional research is needed to further investigate factors that predict optimal mental health and thriving. Consistent with previous research [10-12], the participants were experiencing a high degree of psychological distress. It is well established that psychological distress has both direct and indirect effects on physical health. Findings underscore a need to focus potential interventions on promoting positive mental health and reducing psychological distress.

The responses were relatively evenly distributed with respect to intuitive eating, weight-related concern, and emotional eating. Education around developing a health-promoting relationship with eating may be helpful. This finding supports the utility of, for example, adding nutritional consultation to state Adult Autism Medicaid Waivers [32-34]. Because Medicaid waivers are among the limited insurance options for emerging adults that extend into older adulthood, targeting these interventions with Medicaid waivers may especially support meeting the goal of increased access. The respondents’ physical activity patterns aligned with recommendations from federal guidelines [32], and they generally reported that they exercise to be healthy and feel good. These positive findings suggest that physical activity education may not be a high priority for this population, yet opportunities to enforce these capacities may be warranted.

Self-reported health needs

Areas of high need spanned several domains. The first pertained to buying and cooking healthy food on a budget and practicing healthy nutrition. Taken together with findings from the health assessment, these findings indicate that this population requires strategies for integrating a health-promoting relationship with food in their lives, such as how to engage in intuitive eating, balance financial costs and health, and practice healthy nutrition on a daily basis. This conclusion aligns with ongoing efforts to support developmentally based health-promoting lifestyle behaviors and knowledge in the population [35].

The second pertained to improving mental health. Consistent with previous research on children [13], the participants mentioned sleep as an area of high need. This finding suggests that sleep may play a role in the mental health challenges related to low flourishing and high psychological distress identified in this research. Thus, potential interventions should consider sleep hygiene as a potential mechanism underlying poor mental health and provide support in this area.

The third pertained to navigating the healthcare system. The respondents expressed low confidence and high desire to learn about different health insurance coverage options, the specific details about their health insurance, and finding a doctor. Potential interventions should provide guidance surrounding navigating healthcare and services. The intervention should feature the perspectives of experts in the healthcare sphere to correct any misinformation that this population may have due to receiving health and healthcare information from a range of sources. Efforts can be informed by toolkits developed by self-advocates [36].

The needs assessment also asked about learning preferences for a potential virtual intervention. They reported preferences for a range of virtual accommodations and features, which is consistent with findings on diverse learning preferences in neurodivergent and neurotypical emerging adult populations [37].

This research coalesced into the following recommendations for a potential intervention: 1) Mental health efforts should focus on cultivating strategies to promote positive mental health and reduce psychological distress. Strategies may include cultivating sleep hygiene. Efforts can also focus on promoting optimal health, well-being, and thriving, potentially using strategies, such as mindfulness, meditation, and others [38]. 2) Focus physical health content on developing a health-promoting relationship with food by cultivating intuitive eating, reducing weight-related concerns, and understanding potential emotional eating patterns. Practical information about buying food on a budget should be prioritized. 3) Provide education and practical support surrounding navigating the healthcare system and services. 4) Prioritize a pedagogical approach that is accessible and multimodal and integrates perspectives from interdisciplinary experts in the field.

Based on these recommendations, we developed an initial blueprint for “Adulting for Health,” a potential virtual intervention program to address the priorities identified in this research (see Appendix).

Limitations and future directions

The current study has several limitations. This research relied on a small sample size with intermittent missing data on certain survey sections. Although this approach was acceptable for an exploratory and descriptive study, future research should survey larger samples of neurodivergent emerging adults and hone in on the specific needs, experiences, and priorities of individuals with specific conditions (e.g., autism) given the high degree of heterogeneity inherent in this population. In addition, we prioritized the collection of specific health information to minimize participant burden from surveys. More comprehensive health assessments should be conducted in further efforts to develop intervention curricula. As an example, the physical health domains included in our assessment were selected based on physical health challenges experienced by the general emerging adult population that are emphasized in the literature but were not exhaustive of the myriad physical health issues that may be relevant for this population. Generally, this research was exploratory and meant to spur additional research in this area. We propose that future research replicates and expands the current study.

## Conclusions

The current study captured the health and healthcare needs of neurodivergent emerging adults in order to inform interventions to ameliorate their health disparities. Potential future intervention approaches should focus on specific high-need areas of mental and physical health, support participants in navigating healthcare, and adopt an approach that prioritizes accessibility and interdisciplinary healthcare perspectives. In presenting the conceptualization of a potential virtual intervention program based on these results-“Adulting for Health” -we aim to spur additional development and testing of promising practices that adapt developmentally-based and neurodiversity-oriented approaches.

## Appendices

Appendix A 

**Table 5:** “Adulting for Health” curriculum (structure and approach) ^1^This approach contrasts with approaches that ascertain diagnosis utilizing validated screeners and exclude participants based on results. Given the paucity of research in this area—as well as the long history of excluding neurodivergent individuals from research based on arbitrary criteria [39]—additional research is warranted before narrowing the inclusion criteria. ^2^Technology-mediated interventions for neurodivergent individuals are vastly heterogeneous with respect to duration [40], and no specific duration is, to date, associated with a greater level of efficacy [41]. ^3^This aligns with the approaches from other developmentally based virtual intervention programs [27].

Inclusion criteria	Any individual who identifies with any neurodivergent condition will be eligible to participate^1^
Structure	Intervention sessions will include approximately one hour per week of live pedagogy, accompanied by ad-hoc out-of-class assignments, activities, and opportunities for skill-building. Initial iteration will be a six-week program based on the number of high-need areas we identified as a part of this research.^2^
Approach	Each session and activity will utilize multiple teaching and presenting modalities (i.e., PowerPoint presentations, videos/multimedia, readings for diverse audiences, discussions, and reflections) and will integrate visual, verbal, and auditory approaches. Content will be delivered by interdisciplinary healthcare professional teams (e.g., pediatricians, primary care providers, psychologists, social workers, and others) to account for participants’ multidisciplinary health and healthcare needs.^3^ Teams will include healthcare professionals and trainees that identify as neurodivergent or have specific lived experience in order to cultivate additional engagement from intervention participants.

Appendix B 

**Table 6:** “Adulting for Health” curriculum (content) ^1^These themes will be integrated in all aspects of the intervention based on the well-established research base that finds these skills to be important for health.

Focal areas	Specific topics	Didactic content covered	Skills building and practice	Example discussion and reflection
Week 1: Goal-Setting	The importance of working toward individualized and tailored health goals	What are SMART Goals?	Developing SMART Goals for mental and physical health and healthcare	How can you make your goals more motivating and achievable?
Week 2: Developing a Health-Promoting Relationship with Nutrition	The role of nutrition in health	What should a balanced dinner plate look like?	Engaging in a virtual grocery shopping simulation to practice shopping for a balanced plate	What is a nutritionally balanced dinner you can prepare this week?
Week 3: Developing a Health-Promoting Relationship with Physical Activity	The role of physical activity and exercise in health	What should physical activity look like on a daily and weekly basis to promote health?	Identifying opportunities for physical activity in daily life	What types of physical activity do you enjoy?
Week 4: Navigating Mental Health Challenges	Mental health challenges commonly experienced by emerging adults	What are key resources and supports for anxiety, depression, sleep issues, and other conditions?	Developing an individual mental health toolkit for coping with individual challenges	What is one key resource you plan to use for your mental health?
Week 5: Getting the Healthcare You Need	Common healthcare transition issues for emerging adults	What are key considerations for emerging adults in ensuring positive healthcare experiences?	Creating a healthcare roadmap to identify necessary next steps in securing healthcare services and supports	What are key questions you need to ask your doctor at your next appointment?
Week 6: Positive Mental Health and Thriving	The role of mindfulness, meditation, and civic engagement in health	How can you begin to engage in these activities in daily life?	Practicing mindfulness	How was your experience in our mindfulness activity and how might this skill have a role in your daily life?
Cross-Cutting Themes	Goal-setting and self-advocacy capacities^1^			
